# Efficacy of interventions in peripheral arterial and venous malformations of the limbs in children: protocol for a systematic review

**DOI:** 10.3389/fped.2026.1818097

**Published:** 2026-05-08

**Authors:** Zhaokun Guo, Zhongjing Zhang, Ying Jiang, Jun Zhou, Ke-Heng Deng, Wen-Xiang Zhu, Xiangyou Zhao, Pan Jiao

**Affiliations:** Department of Pediatric Surgery, The First Affiliated Hospital of China Three Gorges University (Yichang Central People's Hospital), Yichang, China

**Keywords:** children, embolism, limbs, peripheral arteriovenous malformations, systematic review

## Abstract

**Background:**

Peripheral arteriovenous malformations (pAVMs) of the limbs are common in children, with distinct clinical features and high intervention difficulty. Existing research on its interventions is controversial due to non-standardized criteria, small-sample uncontrolled studies and inconsistent efficacy evaluation, leaving clinicians without high-quality evidence-based guidance.

**Methods and analysis:**

This study will conduct a PROSPERO-registered systematic review, searching PubMed, Embase and other databases for relevant clinical studies. Two researchers will independently screen literature, extract data and assess evidence quality via GRADE. RevMan 5.4 will be used for qualitative and quantitative statistical analysis, with subgroup and sensitivity analyses to explore heterogeneity. Primary outcome is short-term symptom improvement rate within 3 months post-intervention; secondary outcomes include adverse event incidence and quality of life.

**Ethics and dissemination:**

Ethical approval is not required because this study is a secondary analysis of existing data. We will disseminate the findings through peer-reviewed publications.

**Clinical Trial Registration:**

PROSPERO (CRD420261326988).

## Introduction

Peripheral arteriovenous malformations (pAVMs) are congenital vascular anomalies defined by abnormal direct communications between arteries and veins. Pathologically, the vessel walls of these malformations lack normal smooth muscle layers and elastic fibers, which induces hemodynamic disorders and abnormal local tissue perfusion (1–3). As a complex vascular disease, pAVMs have become increasingly important in pediatric surgical practice (1–5). Among pediatric pAVMs, the extremities are the second most common site after the head and neck, accounting for roughly 35%–45% of all cases (1–5).

Pediatric patients are in a critical growth and development period, so extremity pAVMs in children exhibit distinct clinical features compared with those in adults. On the one hand, the nidus of malformed vessels expands and invades surrounding tissues synchronously with the child's growth, which not only impairs the appearance of local soft tissues but also may compress peripheral nerves, blood vessels, and bones (6–9). This compression can cause limb pain, numbness, and limited mobility; in severe cases, it may lead to limb hypertrophy or atrophy, joint deformities, and even congestive heart failure due to long-term hemodynamic abnormalities, seriously affecting the child's growth, development, and quality of life. On the other hand, children have thinner vessel walls, immature vascular networks, and frequent extremity movements, which increase the difficulty of intervention and the risk of complications (10–13). In addition, parents have high expectations for treatment outcomes, long-term prognosis, and limb function preservation, further complicating clinical management.

Currently, multiple clinical interventions are available for pediatric extremity pAVMs. Common treatment methods in pediatric surgery include surgical resection, endovascular embolization, sclerotherapy, and multidisciplinary comprehensive interventions (e.g., embolization combined with surgery or sclerotherapy combined with surgery) (14–17). Among these, endovascular embolization has become the first-line intervention for pediatric extremity pAVMs due to its minimal invasiveness and limited impact on limb function. Surgical resection is mainly suitable for cases with localized, well-demarcated nidi that do not involve major neurovascular structures; although this method can completely remove the malformed vascular mass, it is more invasive and may cause postoperative complications such as scarring and limb dysfunction (14–19). Sclerotherapy is often used as an adjuvant treatment for small, diffuse lesions, where chemical agents are used to damage the endothelial cells of malformed vessels, resulting in vascular occlusion.

Despite continuous progress in clinical intervention techniques, research on the efficacy of treatments for pediatric extremity pAVMs remains controversial and inadequate. First, although the incidence of pediatric extremity pAVMs is not extremely low, significant individual differences—including variations in nidus size, location, involvement range, and hemodynamic characteristics—have led to the lack of standardized criteria for intervention selection in existing studies, as well as substantial heterogeneity in efficacy evaluation indicators, making direct comparisons between studies difficult (3–7). Second, most existing studies are retrospective case series with small sample sizes (usually 5–30 cases), and most lack control groups, belonging to uncontrolled studies. Only a few are small-sample non-randomized controlled studies, and high-quality randomized controlled trials (RCTs) are notably absent (6–10). As a result, the reliability and reproducibility of research results are poor, with a high risk of bias. Third, the criteria for evaluating intervention efficacy vary across studies: some focus only on short-term symptomatic improvement, while others emphasize the nidus occlusion rate. Few studies have conducted long-term follow-up on limb function, impacts on growth and development, or complication recurrence, and follow-up durations vary widely (3 months to 5 years), further increasing the heterogeneity of results (13–18). When selecting intervention strategies, clinicians lack guidance from high-quality evidence-based medicine and often rely on personal clinical experience, leading to significant differences in treatment choices among different medical institutions and individual physicians. Consequently, some pediatric patients may suffer from increased complications or malformation recurrence due to inappropriate treatment plans, which may even affect normal limb growth and development.

In view of the above clinical and research status, this study intends to conduct a systematic review of the efficacy of interventions for pediatric extremity pAVMs. We will comprehensively search relevant domestic and international clinical studies, systematically analyze the efficacy and safety of various interventions, and identify factors affecting treatment outcomes and complications. Additionally, we will objectively evaluate the risk of bias and evidence quality of existing studies. The results of this study are expected to provide a reliable evidence-based basis for pediatric surgeons to select optimal intervention strategies and formulate standardized diagnosis and treatment guidelines for pediatric extremity pAVMs, as well as offer directions and references for future high-quality clinical research in this field. This systematic review aims to address the following research question based on the PICOS framework: Among children with peripheral arteriovenous malformations of the limbs (Population), what is the efficacy and safety of various interventions including endovascular embolization, surgical resection, sclerotherapy, and combined approaches (Intervention) compared with alternative interventions or conservative management (Comparison) in terms of short-term symptom improvement rate, adverse events, and quality of life (Outcomes)?

## Methods

### Protocol and registration

The protocol for this systematic review has been registered with the International Prospective Register of Systematic Reviews (PROSPERO) under registration number CRD420261326988 (20). This systematic review will be conducted in accordance with the Preferred Reporting Items for Systematic Reviews and Meta-Analyses (PRISMA) and PRISMA-P guidelines (21). Following registration, the review will be conducted strictly in accordance with the registered protocol. Should any adjustments to the inclusion/exclusion criteria, outcome measures, or other aspects be necessary during the research process, these updates will be documented on the PROSPERO platform. The final report will provide a detailed explanation of the reasons for the adjustments, the specific changes made, and the timing of these modifications, thereby ensuring research transparency and reproducibility while minimizing publication bias and selective reporting bias.

### Eligibility and exclusion criteria

#### Study type

Given the scarcity of randomized controlled trials and high-level evidence in the field of pediatric extremity arteriovenous malformations, inclusion of uncontrolled case series is necessary to capture the full spectrum of available clinical data and provide a comprehensive evidence synthesis. All clinical studies related to the efficacy of interventions for pediatric peripheral arteriovenous malformations of the extremities will be included, encompassing uncontrolled case series, retrospective cohort studies, prospective cohort studies, non-randomized controlled studies (non-RCTs), and randomized controlled trials (RCTs). Exclusion criteria will include pure basic research (such as cell experiments, animal experiments, and *in vitro* studies), reviews, commentaries, conference abstracts, letters, editorials, and publications with incomplete data or from which valid information cannot be extracted. Duplicate publications will be excluded, retaining only the one with the most recent publication date and the most complete data.

#### Participants

Included study subjects will be children and adolescents aged ≤18 years with a confirmed diagnosis of peripheral arteriovenous malformations of the extremities based on imaging examinations [such as color Doppler ultrasound, computed tomography angiography (CTA), magnetic resonance angiography (MRA), digital subtraction angiography (DSA), etc.] or pathological examination. The malformation must be clearly located in the extremities (upper limb: palm, dorsum of hand, wrist, forearm, elbow, upper arm, peripheral axilla; lower limb: sole, dorsum of foot, ankle, calf, peripheral knee, thigh, peripheral groin). Arteriovenous malformations of the central nervous system, head and neck, trunk, peripheral viscera, and bone parenchyma will be excluded. Patients with other severe congenital diseases (such as congenital heart disease, coagulation disorders, immunodeficiency, etc.), malignant tumors, other vascular malformations (such as venous malformations, lymphatic malformations, etc.), and those unable to cooperate with treatment or follow-up will also be excluded.

#### Intervention

Any clinical interventions employed in the included studies for pediatric extremity pAVMs will be considered. These interventions may be used alone or in combination and primarily include the following categories: (1) endovascular interventional embolization; (2) surgical treatment; (3) sclerotherapy; (4) multidisciplinary comprehensive interventions: the combined use of two or more of the above intervention modalities; and (5) other interventional approaches. For uncontrolled studies, specifying the intervention is sufficient. For controlled studies (RCTs, non-RCTs, cohort studies), different interventions may be used in the experimental and control groups, or different dosages, treatment courses, or operational protocols of the same intervention may be employed. All such studies will be included, with no restrictions placed on the type of control measures.

#### Comparison

For uncontrolled studies, no control group is required; only the efficacy and safety of the single intervention will be analyzed. For controlled studies (RCTs, non-RCTs, cohort studies), control measures may include comparisons between different interventions (e.g., endovascular embolization vs. surgical treatment, endovascular embolization vs. sclerotherapy), or intervention vs. conservative management (e.g., watchful waiting, symptomatic supportive care). If the control measure involves a placebo, the implementation of blinding must be carefully assessed during the risk of bias evaluation. Studies will be excluded if the control measures are not clearly described or are deemed inappropriate (e.g., lacking clinical comparability with the intervention).

### Outcomes

#### Primary outcome

Short-term symptom improvement rate: The improvement of clinical symptoms such as pain, numbness, swelling, and limited mobility in the extremities of pediatric patients after intervention. Outcomes are categorized as complete remission, partial remission, or no remission. Complete remission is defined as the complete resolution of clinical symptoms, enabling the child to resume normal activities. Partial remission is defined as significant alleviation of clinical symptoms without interfering with daily activities. No remission is defined as no improvement or even worsening of clinical symptoms. The complete remission rate and the overall symptom improvement rate (complete remission + partial remission) will be calculated. The follow-up period is set at within 3 months post-intervention (short-term).

#### Secondary outcomes

Incidence of adverse events, quality of life.

### Exclusion criteria

In addition to the exclusion criteria specified in the inclusion criteria above, the following studies will be additionally excluded: (1) studies where the subjects are adults (age >18 years), or where children and adults are mixed and data for children cannot be extracted separately; (2) studies where the malformation location is unclear, or where pAVMs in sites other than the extremities are included and data for the extremities cannot be extracted separately; (3) studies where the intervention measures are unclear, or where the intervention protocol (e.g., embolic materials, surgical approach, type and dosage of sclerosant) is not described in detail; (4) studies with incomplete outcome data, where valid data cannot be obtained even after contacting the authors; (5) duplicate publications, plagiarized articles, and studies of very poor quality (e.g., lacking standardized diagnostic criteria or clearly defined outcome evaluation methods); (6) studies not published in English (due to language limitations of the researchers in accurately extracting data and to avoid language bias).

### Literature sources and retrieval strategy

A systematic literature search will be designed and executed by an experienced medical librarian. The following electronic databases will be: PubMed, Embase, Cochrane Library, and Web of Science. The search timeframe for all databases will be set from the inception of each database to the date of retrieval, without any time restrictions, to ensure comprehensiveness of the search and avoid missing early literature. A detailed search strategy has been developed. For example, the PubMed search string will be: ["Arteriovenous Malformations"[Mesh] OR "arteriovenous malformation"[tiab] OR "AVM"[tiab]] AND ("Extremities"[Mesh] OR "limb"[tiab] OR "upper extremity"[tiab] OR "lower extremity"[tiab]) AND ("Child"[Mesh] OR "pediatric"[tiab] OR "children"[tiab] OR "adolescent"[tiab]) AND ("Therapeutics"[Mesh] OR "embolization"[tiab] OR "sclerotherapy"[tiab] OR "surgery"[tiab] OR "intervention"[tiab]).

### Literature screening and data extraction

Literature screening will be independently conducted by two systematically trained researchers (both pediatric surgeons or healthcare professionals familiar with vascular malformation-related knowledge and systematic review methodology) strictly according to the pre-defined inclusion and exclusion criteria. The specific process is as follows: (1) Initial screening: Titles and abstracts of the retrieved articles will be read to exclude those that clearly do not meet the inclusion criteria (such as basic research, reviews, studies with inappropriate malformation locations), while retaining articles that potentially meet the criteria. (2) Full-text review: The full text of the articles retained after initial screening will be obtained and carefully reviewed, including the study subjects, interventions, control measures, outcome indicators, and data completeness, to further select studies meeting the inclusion criteria and exclude those that do not. (3) Disagreement resolution: If disagreements arise between the two researchers during the screening process, they will first attempt to resolve them through discussion. If consensus cannot be reached, a third senior pediatric surgeon (familiar with evidence-based medicine and systematic reviews) will be consulted to make the final judgment on whether to include the study. (4) Literature management: EndNote X9 software will be used to manage all retrieved literature, including import, deduplication, and screening markings. During deduplication, the publication with the most recent date and the most complete data will be preferentially retained, while duplicate publications will be excluded.

For the finally included studies, two researchers will independently extract data using a pre-designed data extraction form. After extraction, the results will be cross-checked. If inconsistencies in the data arise, the original articles will be consulted promptly for correction, and the authors will be contacted to obtain supplementary data if necessary. The data extraction form is designed based on the PICOS elements and outcome indicators of this study, encompassing the following aspects: (1) Basic study information: study title, first author, publication year, journal, study type (uncontrolled case series, cohort study, non-RCT, RCT), sample size, follow-up duration, and study region/country. (2) Baseline information of study subjects: age of pediatric patients (mean age, age range), sex ratio, malformation location (upper/lower limb, specific site), malformation size, disease duration, diagnostic criteria, and inclusion/exclusion criteria. (3) Intervention details: type of intervention (e.g., embolization, surgery, sclerotherapy). (4) Control measure details (for controlled studies only): intervention type and specific protocol in the control group, number of interventions, and treatment course. (5) Outcome indicator data, etc.

### Certainty of evidence assessment

Study-level risk of bias will be assessed using the Cochrane Risk of Bias Tool for randomized controlled trials, the ROBINS-I tool for non-randomized studies, and the NIH Quality Assessment Tool for Case Series. The overall certainty of evidence will then be evaluated using the GRADE approach (22).

### Statistical analysis

Data synthesis will be performed using a combination of qualitative description and quantitative analysis, conducted independently by two researchers. Statistical analysis will be carried out using RevMan 5.4 software. If the included studies involve the same intervention and the same outcome indicators, and demonstrate homogeneity in study design, baseline characteristics of study subjects, and outcome assessment methods (no significant heterogeneity), a quantitative Meta-analysis will be attempted for pooled estimation. Heterogeneity will be assessed using the chi-square test (*χ*^2^) combined with the I^2^ statistic, with a significance level set at *α* = 0.10. Heterogeneity is interpreted as follows: I^2^ < 25% indicates no significant heterogeneity; 25%-50% indicates mild heterogeneity; 51%–75% indicates moderate heterogeneity; and >75% indicates high heterogeneity. In cases of mild to moderate heterogeneity, a random-effects model will be used for quantitative pooling, and sources of heterogeneity (such as patient age, severity of malformation, intervention protocol) will be explored. If high heterogeneity is present, and the source cannot be identified or reduced through subgroup analysis or sensitivity analysis, quantitative Meta-analysis will be abandoned, and only qualitative description will be performed.

For uncontrolled case series, the focus will be on analyzing the range of incidence rates and the average incidence rate for each outcome indicator under the same intervention. Descriptive statistics will be used for summarization, without the need for inter-group heterogeneity testing or quantitative pooling. For dichotomous outcome variables, the odds ratio (OR) will be used as the effect measure, along with its 95% confidence interval (95% CI). For uncontrolled studies, only the incidence rate and 95% CI for each study will be calculated, and the pooled average incidence rate will be presented. For controlled studies, the OR and 95% CI between the experimental and control groups will be calculated to compare the differences in outcome indicators. For continuous outcome variables, the standardized mean difference (SMD) will be used as the effect measure, with its 95% CI calculated.

If the number of included studies for a specific intervention and outcome indicator is ≥10, publication bias will be assessed. Funnel plot symmetry will be visually inspected, supplemented by Egger's test and Begg's test for quantitative verification (significance level *α* = 0.05). Symmetrical funnel plot with *P* > 0.05 for both tests suggests no significant publication bias. Asymmetrical funnel plot with *P* < 0.05 indicates publication bias; the trim-and-fill method will be used for correction, and the impact and potential sources of bias will be discussed. If the number of included studies is <10, publication bias testing will not be performed, and the potential risk of bias will be addressed in the discussion.

### Subgroup and sensitivity analyses

Subgroup analyses will be conducted to explore potential sources of heterogeneity and to identify factors affecting treatment outcomes and complications. If a sufficient number of studies are available (≥5 studies for the same intervention) and significant heterogeneity is present, subgroup analyses will be conducted to explore potential sources of heterogeneity and further clarify differences in intervention efficacy across subgroups. The predefined subgroups are as follows: (1) Age subgroup: ≤10 years vs. 11–18 years; (2) Malformation location subgroup: upper limb vs. lower limb; (3) Severity subgroup (based on reported size and extent): mild vs. moderate-to-severe; (4) Intervention protocol subgroup: different embolic agents, surgical techniques, sclerosants, and single vs. multiple sessions; (5) Study quality subgroup: high-quality vs. moderate-quality studies. Subgroup analyses will employ the same statistical methods as the overall analysis. If heterogeneity is reduced within a subgroup, quantitative pooling will be performed; if substantial heterogeneity remains, only qualitative description will be provided.

To verify the stability and reliability of the conclusions, sensitivity analysis will be performed if Meta-analysis is conducted: (1) Studies with small sample sizes (e.g., <10 cases) will be excluded, and the analysis will be repeated; (2) The statistical model will be changed (fixed-effects vs. random-effects), and the pooled effect sizes before and after the change will be compared. If the pooled effect size remains essentially unchanged (95% CI without significant crossover), the conclusions are considered stable and reliable; if a significant change occurs, the conclusions are considered unstable.

## Discussion

This study will focus on the efficacy of interventions for pediatric peripheral arteriovenous malformations of the extremities. Through systematic literature retrieval and evidence synthesis, the clinical value of different interventional approaches will be objectively analyzed, aiming to provide evidence-based guidance for pediatric surgical practice. The strengths of this study include: a precise focus on the extremities, aligning with the clinical needs of pediatric surgery and minimizing heterogeneity associated with multi-site studies; adaptation to the current research landscape by not mandating controlled studies and employing tailored tools to assess the quality of uncontrolled case series, ensuring comprehensive evidence; outcome measures that encompass efficacy, safety, and long-term prognosis, meeting the need for preserving limb function in children; and strict adherence to systematic review protocols, with bias controlled through registration and dual-reviewer operations, enhancing the reliability of conclusions.

This study will have several anticipated limitations: most of the included studies maybe small-sample uncontrolled case series with high heterogeneity and risk of bias; only English-language literature will be included, which may introduce language and publication bias; incomplete data in some studies may affect the accuracy of the analysis; and the uncontrolled designs preclude inter-group comparisons, limiting the generalizability of the conclusions.
